# Hygiene of the Camps and Cantonments of an Army

**Published:** 1918

**Authors:** 


### HYGIENE

***Hygiene of the Camps and Cantonments of an Army.*** By P. Nobécourt, Médecin-Major de 1re classe. Trans. and Abs. from the *Presse médicale*, June, 10, 1918.

The author reviews the progress made in arrangements for comfort and hygiene in camps and cantonments since the outbreak of the war. At first this branch of the service was necessarily neglected in the endeavor to provide adequate hospitalization, but at present hygienic installations are numerous and defective arrangements are rare.

The program for rendering camps and cantonments *clean* and *comfortable* includes the *cleaning* of grounds, buildings, kitchens, and sanitary arrangements; the proper closing, ventilation, and heating of *lodgings*; the *furnishing* of indispensable articles of comfort, the providing of agreeable *meeting places*; the installing of well-kept *infirmaries* and facilities for *personal hygiene*, and the providing of *pure water*.

*Sanitation of the grounds* is one of the first and most difficult requirements. Places are designated for the deposit of manure, refuse, etc., and the accumulation is removed daily, if possible, to a place remote from the camp. To rid the camp of flies, the manure heaps are sprayed with a 3 per cent. solution of cresyl. The grounds should be graded to permit of proper drainage, and all holes and depressions should be filled in. Streams should be cleared, and where necessary canals dug. Special pains should be taken to allow the water from wells, fountains, wash-places, baths, kitchens, etc., to drain off.

The ideal situation for a camp is a gentle slope towards the south or south-east. Camps in a hollow are difficult to maintain in a good state of sanitation.

Roadways should be kept free of mud and dust, and should be sprinkled in the dry seasons. Stone and log roads are constructed for the pack-animals, and paths with proper flagging for the men. Mud is a great enemy, not only because of its filth, but also because of the constant wetting of feet.

Proper arrangements for the disposal of manure and refuse should be made near the cantonment, including an incinerator. They should be so placed that the prevailing wind will carry the odor away from the camp. They should be situated on dry ground, and distant from water supply and streams.

Manure should be placed in regular piles with evaporating canals to prevent the spread of the liquids. In summer the piles should be covered with earth to prevent the increase of flies, or, if this measure is insufficient, they should be sprinkled with a 3 per cent. solution of cresyl or a 10 per cent. solution of iron sulphate (sulfate ferrique).

Things worth saving from the dumps are put aside and treated according to their nature. Old clothes are sprayed with the Vermorel apparatus using a solution of cresyl. Bones are buried in a pit, with light layers of dirt over each deposit.

Organic detritus is deeply buried or burned, the latter process being preferable. Incinerating furnaces are easily constructed with stones or bricks, or by digging into a bank.

If these measures do not suffice to avoid fly and rat pests, other means like poisons and rat-dogs may be employed.

The drying of all ground in the neighborhood of a camp helps to rid the place of mosquitoes and to prevent malaria.

The construction of proper *latrines* is of prime importance. At first, the holes dug were too small, with the results that they were soon filled and the men obliged to defecate elsewhere. They are now dug very deep, and strict discipline is maintained in the care of such places. Disinfectants are freely used, and men are required to cover their stools with layers of dirt. Every effort is made to render them confortable and private. In summer a small quantity of coal-tar mixed with iron sulphate (sulfate ferrique) is spread each day over the deposite to prevent the accumulation of flies. The pits should be covered with earth before they are filled and the superstructure moved to a newly dug trench.

The use of metal receptacles is very awkward and should be resorted to only when the ground is too swampy to permit of pits or when the conditions of service require their use.

The *maintenance of lodgings* is a difficult and varied task, but requires the utmost thoroughness and care. It is difficult to do much in this particular during times of stress, but behind the lines, all places of habitation may, at least, be kept in good condition. Rough buildings like barns, should be improved by the construction of walls and ceilings, and every facility provided for heating and ventilation. Over-crowding should never be permitted and sufficient free space in each place of lodging should be allowed for the men to gather. Fortunately cots and straw mattresses have everywhere replaced the loose straw which in the early days of the war had to serve for bedding even in the rest billets. Of the types of field barracks, the author considers the *baraques Adrian* the best. Such a barracks may accomodate 120 men with plenty of space for eating and lounging The *foyers de soldats*, the Union Franco-Américaine, etc., are of great value in providing other facilities and means of recreation.

Special care is needed in the proper installation of kitchens, even if they are only the rolling kitchens of campaign use. The ground should be paved or covered with cement to permit of cleaning, and proper means of drainage provided.

Another improvement made in the cantonments is the installation of well-kept infirmaries. A delousing chamber for disinfestation by sulphur and a bathing establishment for the treatment of skin diseases are necessary additions.

Personal sanitation has been greatly improved by the installation of shower-baths in most of the camps and cantonments. Bathing is required once a week, but a shower may be taken at any time. A laundry should be added to the bathing establishments to provide a change of underclothes after each weekly bath.

*Sections d'hygiène corporelle* instituted in August 1917, provide these improvements in each army. Forty men may be bathed at the same time, and their clothing disinfected in a steam chamber. The *camions-douches automobiles* which were previously used, serve the same purpose.

The bath alone does not suffice to maintain a sanitary bodily condition. There should also be arrangements permitting the men to wash twice daily. Provision should also be made for the men to wash out articles of clothing which are not received with the regulation garments at the army laundries.

The *water supply* is the object of greatest concern. Bacteriological examination is indispensable. In most places there is an abundant water supply, but it is often in such form as to be easily contaminated. Every precaution should be taken to prevent infiltration of filth. No garbage, manure, latrines, etc., should be allowed in the vicinity. The army water service makes all necessary arrangements in advance. Pumps are installed when possible to prevent the use of dirty dippers.

*Javellization* is resorted to when necessary. The greatest care must be exercised in employing this method. Water so treated in casks may be easily recontaminated. It is often objectionable in taste, and for every reason is less desirable than water not so treated.

Water procured at a distance should, when possible, be conveyed in pipes or canals and not in barrels, which are a frequent source of infection.

A considerable and efficient sanitary corps has grown up to meet the war needs, and the army physicians devote much of their attention to the same ends. The author expresses the hope that this branch of the service may be still greatly increased and that its good effects may be extended to times of peace.

*Le Gérant :* O. Porée. Paris — Imp. Lahure, 9, rue de Fleurus.

